# Supplementary material for: Rare loss of function variants in the hepatokine gene INHBE protect from abdominal obesity
Source: Nat Commun. 2022 Jul 27;13:4319. doi: 10.1038/s41467-022-31757-8 (PMC9329324; doi:10.1038/s41467-022-31757-8)
Supplement: Supplementary file 1 — Supplementary Information [file 41467_2022_31757_MOESM1_ESM.pdf]

Rare loss of function variants in the hepatokine gene *INHBE* protect from abdominal obesity

Aimee M. Deaton[1*], Aditi Dubey[1], Lucas D. Ward[1], Peter Dornbos[2,3,4], Jason Flannick[2,3,4], AMP-T2D-GENES Consortium[**], Elaine Yee[1], Simina Ticau[1], Leila Noetzli[1], Margaret M. Parker[1], Rachel A. Hoffing[1], Carissa Willis[1], Mollie E. Plekan[1], Aaron M. Holleman[1], Gregory Hinkle[1], Kevin Fitzgerald[1], Akshay K. Vaishnaw[1], and Paul Nioi[1]

[1] Alnylam Pharmaceuticals, Cambridge, MA
[2] Programs in Metabolism and Medical & Population Genetics, Broad Institute, Cambridge, MA
[3] Division of Genetics and Genomics, Boston Children's Hospital, Boston, MA
[4] Department of Pediatrics, Harvard Medical School, Boston, MA
* Correspondence to adeaton@alnylam.com
** A full list of consortium members is provided in the Supplementary Information

Supplementary Information

Supplementary Figures

Supplementary Figure 1: Gene-level associations with waist-to-hip ratio adjusted for BMI by variant set

Gene-based burden analysis of WHRadjBMI was performed in 362,679 European ancestry individuals for pLOF, missense and pLOF + missense variant masks using a generalized linear model adjusting for the first 30 PCs of genetic ancestry. Genes significant in the overall analysis ($P \leq 1.05 \times 10^{-6}$; Bonferroni correcting for the number of genes and variant masks tested) are labeled. The dashed line indicates the threshold for statistical significance.

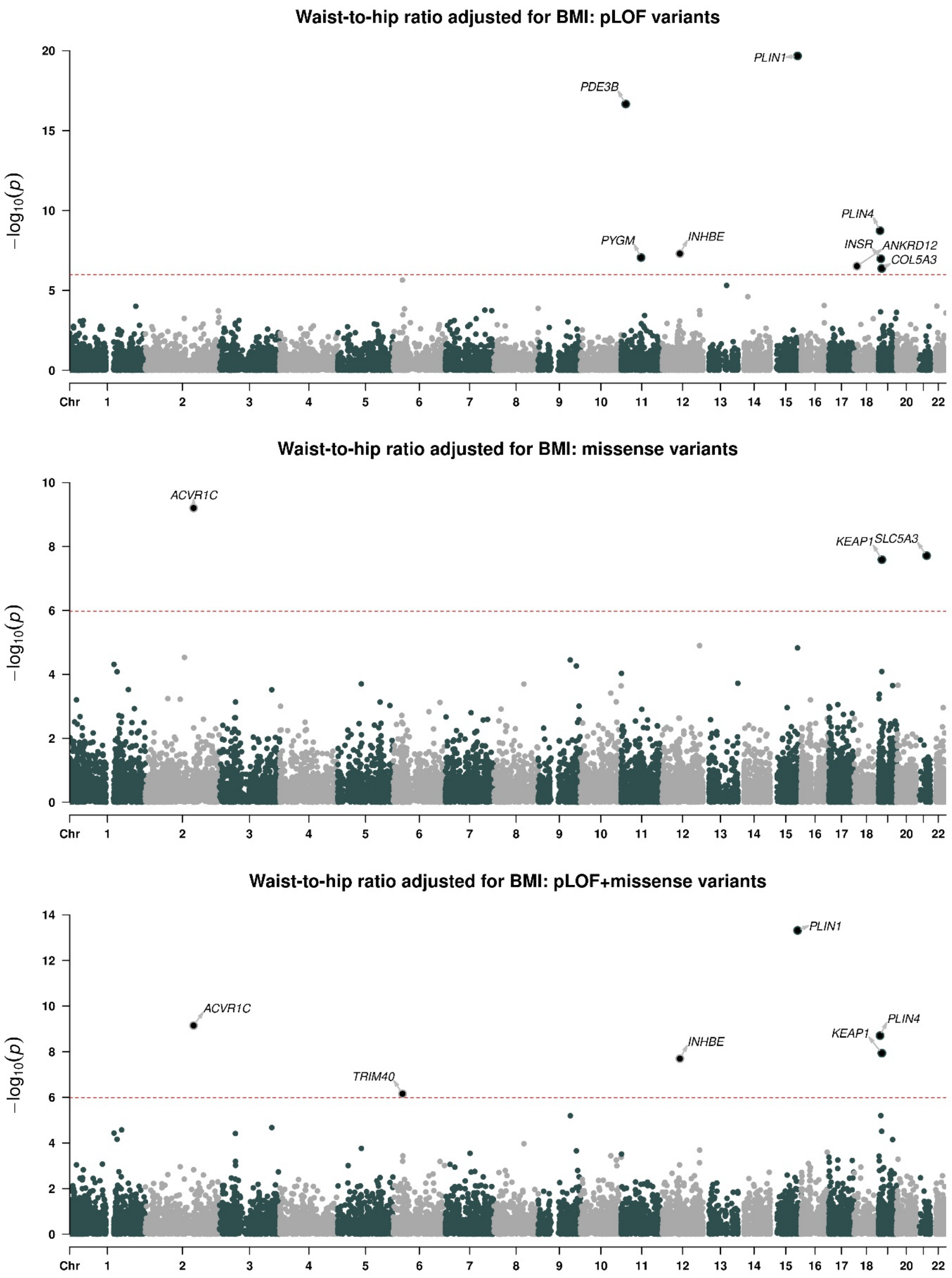

Waist-to-hip ratio adjusted for BMI: pLOF variants
−log10(p)
PLIN1
PDE3B
PLIN4
PYGM
INHBE
INSR
ANKRD12
COL5A3
Chr 1 2 3 4 5 6 7 8 9 10 11 12 13 14 15 16 17 18 20 22
Waist-to-hip ratio adjusted for BMI: missense variants
−log10(p)
ACVR1C
KEAP1
SLC5A3
Chr 1 2 3 4 5 6 7 8 9 10 11 12 13 14 15 16 17 18 20 22
Waist-to-hip ratio adjusted for BMI: pLOF+missense variants
−log10(p)
PLIN1
ACVR1C
PLIN4
KEAP1
INHBE
TRIM40
Chr 1 2 3 4 5 6 7 8 9 10 11 12 13 14 15 16 17 18 20 22

Supplementary Figure 2: QQ plots for sex-combined and sex-stratified analysis of WHRadjBMI

Gene-based burden analysis of WHRadjBMI was performed in 362,679 European ancestry individuals for pLOF, missense and pLOF + missense variant masks using a generalized linear model adjusting for the first 30 PCs of genetic ancestry. Analysis was performed in the entire population (sex-combined) or separately in men and women. N represents the number of rare variant carriers per gene and lambda is the genomic inflation factor.

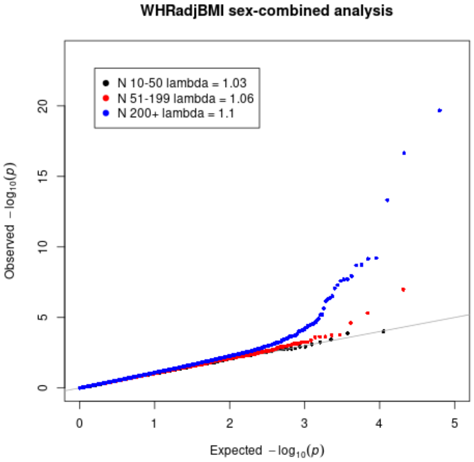

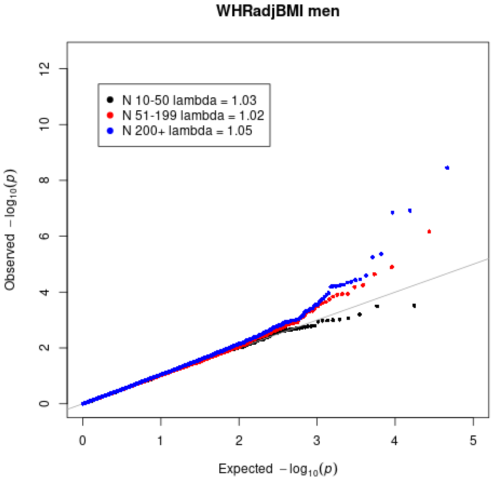

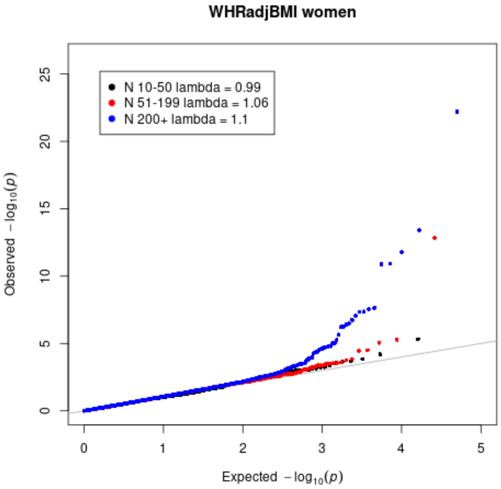

Supplementary Figure 3: Gene-level associations with WHRadjBMI in men

Gene-based burden analysis of WHRadjBMI in men (N = 166,533) was performed for pLOF, missense and pLOF + missense variant masks using a generalized linear model adjusting for the first 30 PCs of genetic ancestry. The best variant set per gene is shown and significant genes are labeled ($P \leq 1.05\text{x}10^{-6}$; Bonferroni correcting for the number of genes and variant masks tested). The dashed line indicates the threshold for statistical significance.

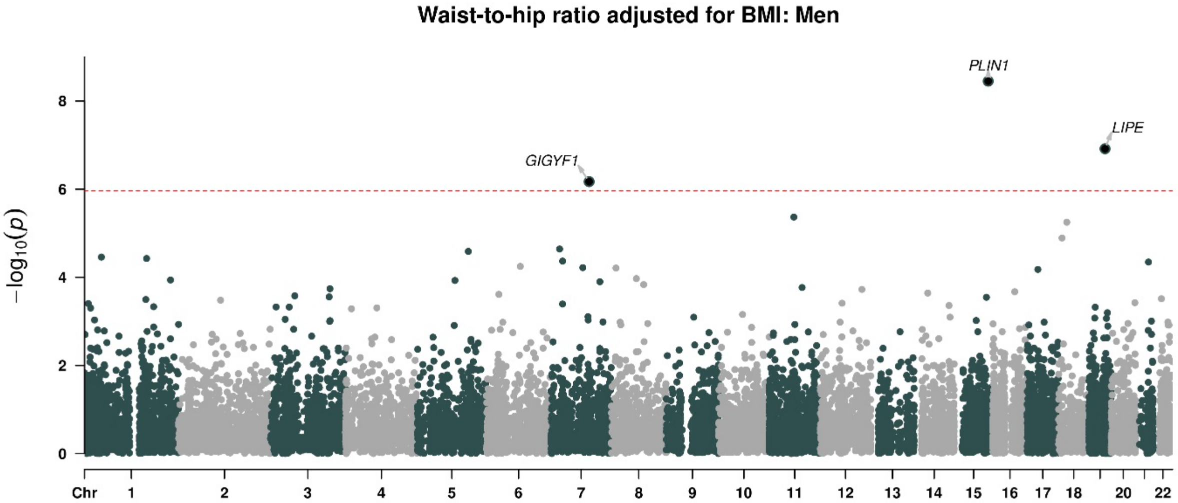

Supplementary Figure 4: Gene-level associations with WHRadjBMI in women

Gene-based burden analysis of WHRadjBMI in women (N = 195,777) was performed for pLOF, missense and pLOF + missense variant masks using a generalized linear model adjusting for the first 30 PCs of genetic ancestry. The best variant set per gene is shown and significant genes are labeled ($P \leq 1.05 \times 10^{-6}$; Bonferroni correcting for the number of genes and variant masks tested). The dashed line indicates the threshold for statistical significance.

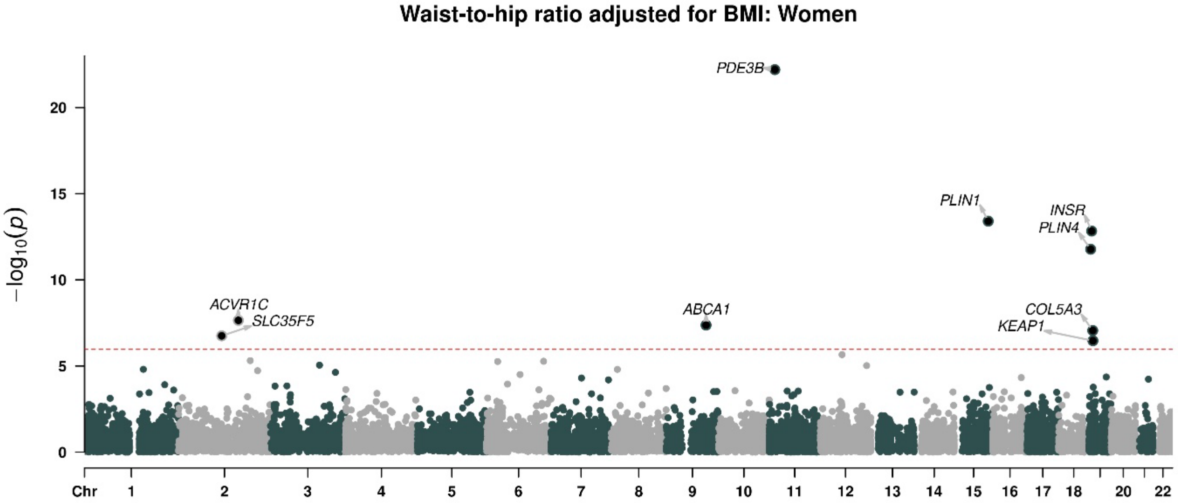

Supplementary Figure 5: Leave-one-variant-out analysis for selected genes

We performed leave-one-variant-out analysis for selected WHRadjBMI-associated genes. Gene-based burden analysis of WHRadjBMI was performed using a generalized linear model excluding one variant at a time from the analysis. The genomic position of the excluded variant is plotted versus -log10(*P*) for the association when that variant is excluded. The variant sets contributing most of the signal in gene-based tests are labeled.

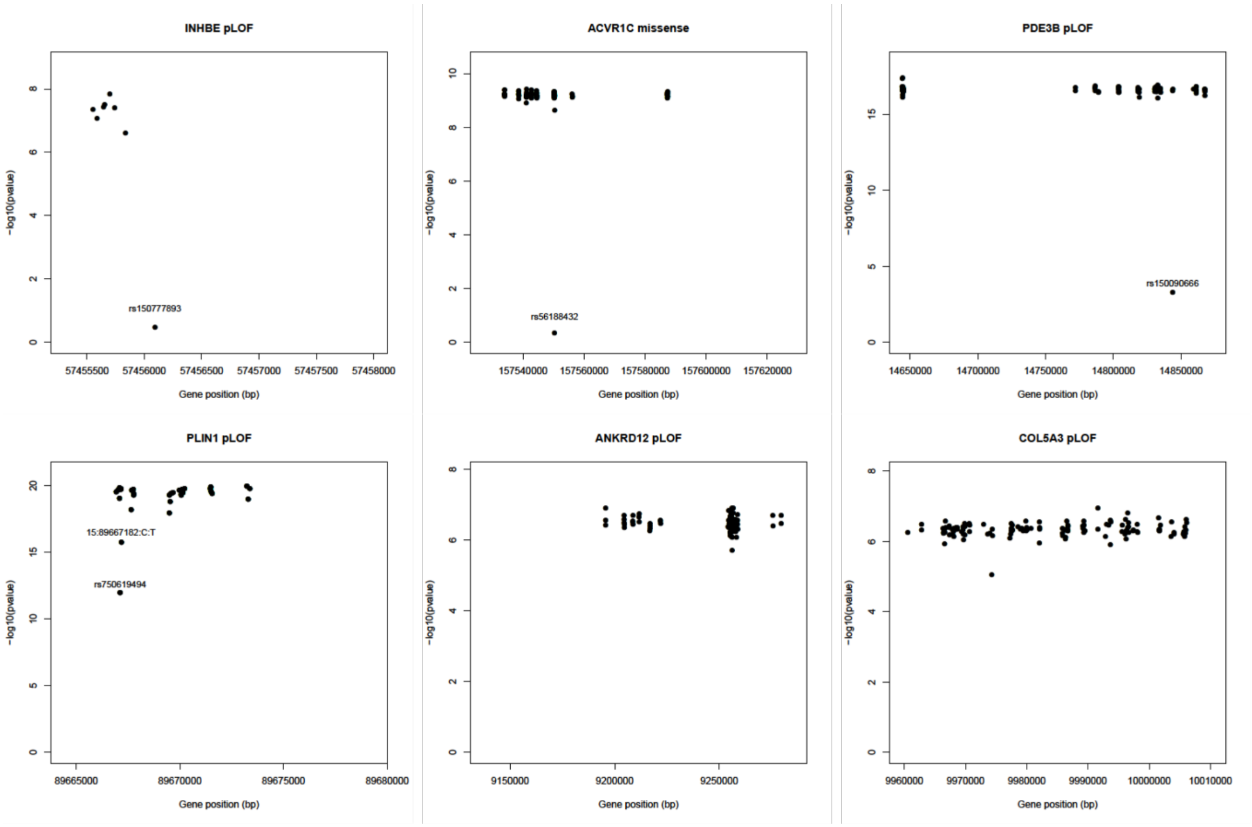

Supplementary Figure 6: Association of significant genes with unadjusted WHR and BMI

For genes associated with WHRadjBMI, we plotted the effect on WHR (without BMI adjustment) compared to the effect on BMI (both in standard deviations; SD) calculated in 362,679 European ancestry participants. Effects are shown for *INHBE* pLOF and, for the other genes, the most significant variant set per gene. Grey bars represent the 95% confidence interval.

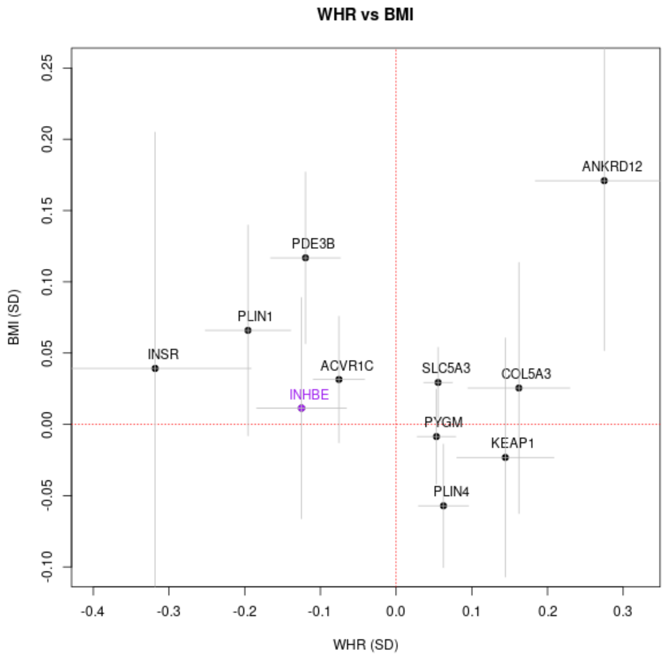

Supplementary Figure 7: Association of significant genes with adipose tissue distribution assessed by MRI

For genes associated with WHRadjBMI, we plotted the effect on WHRadjBMI (tested in 362,679 European ancestry participants) compared to the effect on VATadjBMI and ASATadjBMI (tested in 33,318 European ancestry participants with abdominal MRI). Effects are shown for *INHBE* pLOF and, for the other genes, the most significant variant set per gene. Grey bars represent the 95% confidence interval. *INSR* pLOF is not shown because there were insufficient numbers of carriers with information on adipose distribution assessed by MRI. VATadjBMI, visceral adipose tissue adjusted for BMI; ASATadjBMI, abdominal subcutaneous adipose tissue adjusted for BMI.

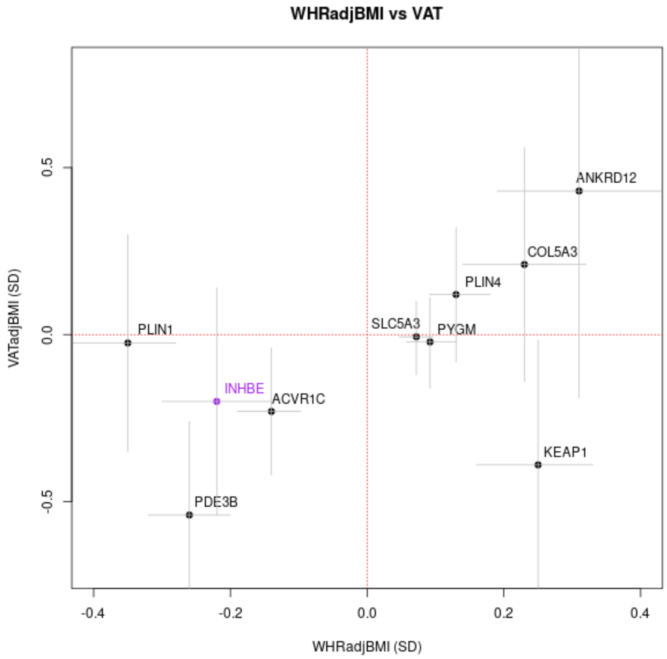
WHRadjBMI vs VAT
VATadjBMI (SD)
WHRadjBMI (SD)
ANKRD12
COL5A3
PLIN4
SLC5A3
PYGM
PLIN1
INHBE
ACVR1C
KEAP1
PDE3B
0.5
0.0
-0.5
-0.4
-0.2
0.0
0.2
0.4

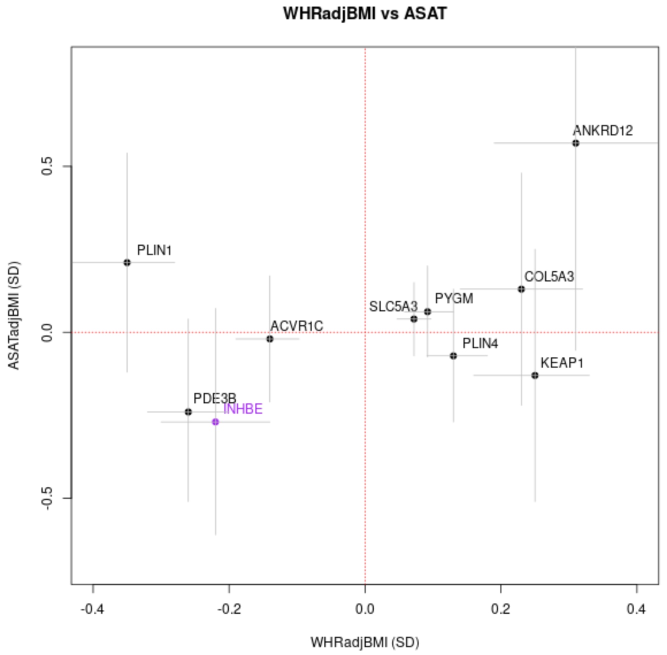
WHRadjBMI vs ASAT
ASATadjBMI (SD)
WHRadjBMI (SD)
ANKRD12
PLIN1
COL5A3
PYGM
SLC5A3
ACVR1C
PLIN4
KEAP1
PDE3B
INHBE
0.5
0.0
-0.5
-0.4
-0.2
0.0
0.2
0.4

Supplementary Figure 8: Survival analysis of *INHBE* pLOF carriers vs non-carriers in UKB

The effect of *INHBE* pLOF genotype on survival was examined using a Cox proportional hazards regression looking at time from enrollment to death in UKB adjusting for age at enrollment, sex and 30 PCs of genetic ancestry.

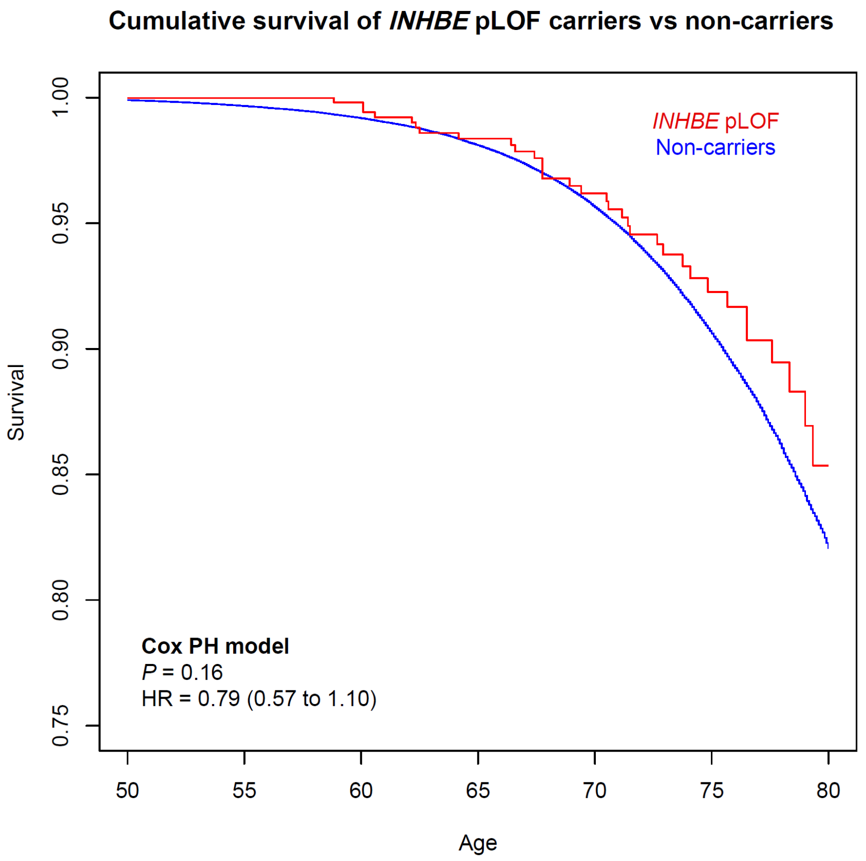

Supplementary Figure 9: Inhibin βE cross-species protein alignment

The DSTS residues deleted by the splice acceptor variant are highlighted.

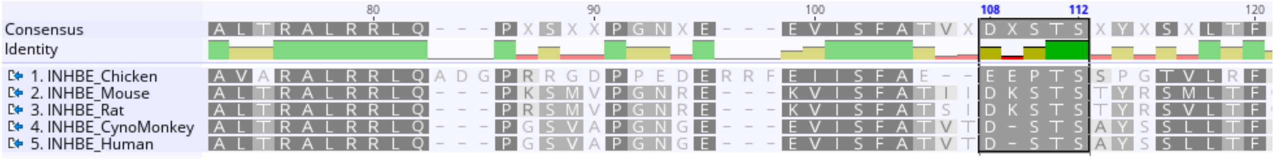

Supplementary Figure 10: Expression of *INHBE* in obese cynomolgus monkeys

Expression of *INHBE* mRNA in the liver of lean (n=24) and obese (n=13) cynomologous monkeys was quantified by RT-qPCR. *INHBE* expression for each animal was normalized to the geometric mean of *ARL6IP4* and *RPS9* expression. We then calculated the mean expression level in lean animals and expressed fold-change in *INHBE* relative to this value. Statistical significance was assessed using a two-sided unpaired t-test ($P = 1 \times 10^{-12}$). Error bars represent the standard error of the mean for each group.

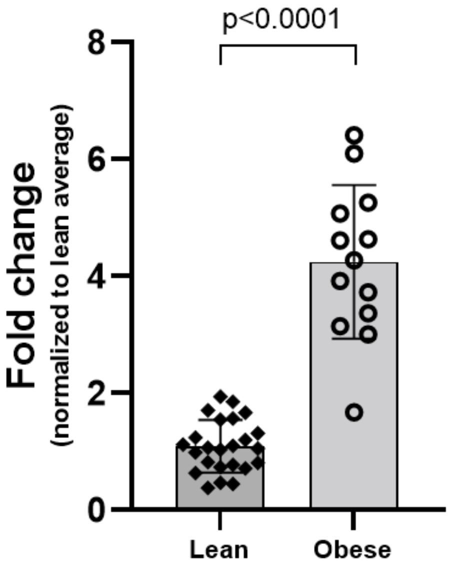

Supplementary Figure 11: WHRadjBMI associations of Mendelian lipodystrophy genes

Gene-based burden analysis of WHRadjBMI in 362,679 European ancestry individuals was performed using a generalized linear model. For genes implicated in lipodystrophies (according to OMIM https://omim.org/), associations with WHRadjBMI are shown for the most significant variant set per gene in the sex combined (All) and sex-stratified analyses. Red lines indicate the threshold for statistical significance ($P \leq 0.004$, Bonferroni correcting for 13 genes tested) and Beta=0.

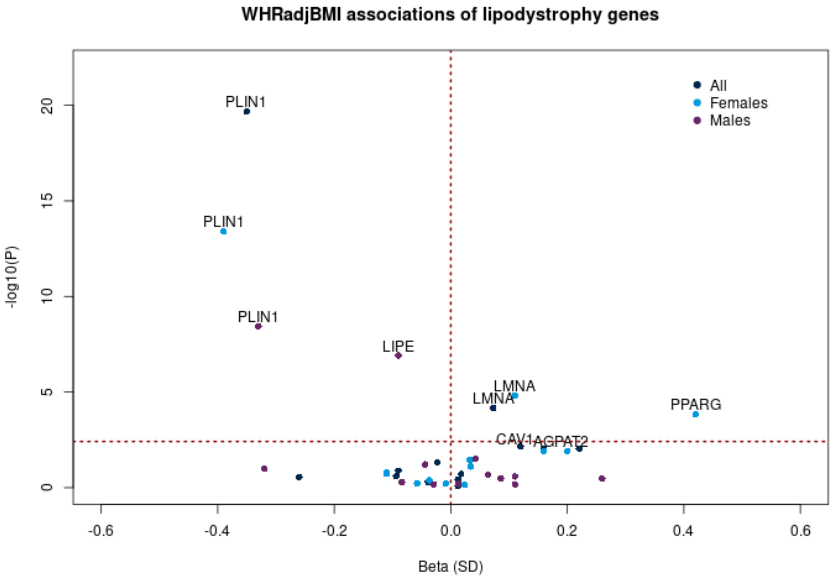

Supplementary Tables

Supplementary Table 1: Gene burden analysis conditioning on nearby hits.

For genes within 1Mb of each other that were significant in the gene burden analysis, namely *KEAP1* and *COL5A3*, we performed conditional analysis. Burden analysis of WHRadjBMI was performed using a generalized linear model adjusting for the first 30 PCs of genetic ancestry and the relevant genotype as indicated ("adjustment"). The threshold for significance was $P \leq 1.05 \times 10^{-6}$ (Bonferroni correcting for the number of genes and variant masks tested).

**Gene**	**Variant set**	**Gene coordinates (hg38)**	**adjustment**	***P***	**Beta (95% CI) in SD units of WHRadjBMI**
COL5A3	pLOF	19:9959561-10010532	unadjusted	4.20E-07	0.23 (0.14, 0.32)
COL5A3	pLOF	19:9959561-10010532	*KEAP1* pLOF+missense	4.03E-07	0.23 (0.14, 0.32)
KEAP1	pLOF+missense	19:10486120-10503378	unadjusted	1.16E-08	0.25 (0.16, 0.33)
KEAP1	pLOF+missense	19:10486120-10503378	*COL5A3* pLOF	1.11E-08	0.25 (0.16, 0.33)
KEAP1	missense	19:10486120-10503378	unadjusted	2.58E-08	0.25 (0.16, 0.34)
KEAP1	missense	19:10486120-10503378	*COL5A3* pLOF	2.48E-08	0.25 (0.16, 0.34)

Supplementary Table 2: Gene burden analysis conditioning on nearby GWAS hits.

We conditioned genes significant in our burden analysis on the top array association(s) with WHRadjBMI (lead SNP in a 250kb window with $P \leq 1.05 \times 10^{-6}$) in a 1Mb window. Conditional analysis was performed on 363,016 European ancestry individuals with exome and array genotypes available, unadjusted values are from our primary analysis. Burden analysis was performed using a generalized linear model adjusting for the first 30 PCs of genetic ancestry and the relevant genotype as indicated ("adjustment"). The threshold for significance was $P \leq 1.05 \times 10^{-6}$ (Bonferroni correcting for the number of genes and variant masks tested in our primary analysis).

**Gene**	**Variant set**	**Adjustment**	***P***	**Beta (95% CI) in SD units of WHRadjBMI**	**N carriers**	**significant**
PDE3B	pLOF	unadjusted	2.17E-17	-0.26 (-0.32, -0.2)	1020	Y
PDE3B	pLOF	rs1037378 and rs76613195	3.91E-16	-0.25 (-0.32, -0.19)	1017	Y
PDE3B	pLOF	rs76613195	3.28E-16	-0.25 (-0.32, -0.19)	1017	Y
PDE3B	pLOF	rs1037378	5.27E-17	-0.26 (-0.32, -0.20)	1017	Y
PLIN4	pLOF	unadjusted	1.84E-09	0.13 (0.091, 0.18)	1961	Y
PLIN4	pLOF	rs13041	1.45E-09	0.14 (0.092, 0.18)	1958	Y
PLIN4	pLOF+missense	unadjusted	1.97E-09	0.13 (0.09, 0.17)	2199	Y
PLIN4	pLOF+missense	rs13041	1.20E-09	0.13 (0.087, 0.17)	2194	Y
INSR	pLOF	unadjusted	1.05E-07	-0.45 (-0.62, -0.29)	135	Y
INSR	pLOF	rs199817986	1.05E-07	-0.45 (-0.62, -0.29)	135	Y
ACVR1C	missense	unadjusted	6.24E-10	-0.14 (-0.19, -0.097)	1892	Y
ACVR1C	missense	rs55920843	6.64E-10	-0.14 (-0.19, -0.096)	1888	Y
ACVR1C	pLOF+missense	unadjusted	7.10E-10	-0.14 (-0.18, -0.095)	1923	Y
ACVR1C	pLOF+missense	rs55920843	7.66E-10	-0.14 (-0.18, -0.095)	1919	Y
SLC5A3	missense	unadjusted	1.93E-08	0.072 (0.047, 0.097)	6141	Y
SLC5A3	missense	rs28451064	1.76E-08	0.072 (0.047, 0.097)	6123	Y
TRIM40	pLOF+missense	unadjusted	6.99E-07	0.074 (0.045, 0.10)	4443	Y
TRIM40	pLOF+missense	rs3094625	2.55E-06	0.07 (0.041, 0.1)	4433	N

Supplementary Table 3: WHRadjBMI associations in men

Results for any variant set significant in the male-specific, female-specific or sex-combined analysis are shown. Burden analysis was performed using a generalized linear model adjusting for the first 30 PCs of genetic ancestry. The threshold for significance was $P \leq 1.05 \times 10^{-6}$ (Bonferroni correcting for the number of genes and variant masks tested). N = 166,533 men with WHRadjBMI measurements.

**Gene**	**Variant set**	**Gene coordinates**	***P***	**Beta (95% CI) in SD units of WHRadjBMI**	**N (carriers|non-carriers)**	**significant**
PLIN1	pLOF	15:89664365-89679417	3.55E-09	-0.33 (-0.44, -0.22)	316|166217	Y
LIPE	pLOF+missense	19:42401507-42427426	1.21E-07	-0.09 (-0.12, -0.057)	3498|163035	Y
LIPE	missense	19:42401507-42427426	1.42E-07	-0.091 (-0.13, -0.057)	3342|163191	Y
GIGYF1	pLOF	7:100679506-100694280	6.76E-07	0.57 (0.34, 0.79)	76|166457	Y
PYGM	pLOF	11:64746389-64760715	4.3E-06	0.12 (0.068, 0.17)	1502|165031	N
ANKRD12	pLOF	18:9136753-9285985	1.28E-05	0.38 (0.21, 0.55)	131|166402	N
SLC5A3	missense	21:34073523-34106262	4.51E-05	0.077 (0.04, 0.11)	2803|163730	N
PLIN1	pLOF+missense	15:89664365-89679417	5.53E-05	-0.087 (-0.13, -0.045)	2140|164393	N
INHBE	pLOF	12:57455291-57458013	3.87E-04	-0.21 (-0.33, -0.095)	277|166256	N
INHBE	pLOF+missense	12:57455291-57458013	0.001	-0.16 (-0.25, -0.062)	410|166123	N
ACVR1C	missense	2:157526767-157628887	0.003	-0.1 (-0.17, -0.033)	857|165676	N
ACVR1C	pLOF+missense	2:157526767-157628887	0.005	-0.096 (-0.16, -0.03)	871|165662	N
KEAP1	pLOF+missense	19:10486120-10503378	0.02	0.15 (0.021, 0.29)	212|166321	N
KEAP1	missense	19:10486120-10503378	0.03	0.15 (0.013, 0.29)	196|166337	N
TRIM40	pLOF+missense	6:30135998-30148773	0.04	0.047 (0.0031, 0.09)	2029|164504	N
SLC35F5	pLOF+missense	2:113702677-113756823	0.14	0.029 (-0.0095, 0.068)	2589|163944	N
SLC35F5	missense	2:113702677-113756823	0.14	0.03 (-0.01, 0.07)	2389|164144	N
COL5A3	pLOF	19:9959561-10010532	0.17	0.093 (-0.039, 0.22)	220|166313	N
PDE3B	pLOF	11:14643691-14874139	0.18	-0.062 (-0.15, 0.028)	464|166069	N

PLIN4	pLOF+missense	19:4502180-4520285	0.38	0.028 (-0.034, 0.089)	1001|165532	N
ABCA1	missense	9:104781002-104928246	0.50	-0.011 (-0.044, 0.021)	3603|162930	N
ABCA1	pLOF+missense	9:104781002-104928246	0.50	-0.011 (-0.043, 0.021)	3804|162729	N
PLIN4	pLOF	19:4502180-4520285	0.61	0.017 (-0.048, 0.083)	888|165645	N
INSR	pLOF+missense	19:7112255-7294405	0.61	-0.019 (-0.095, 0.056)	663|165870	N
INSR	pLOF	19:7112255-7294405	0.67	0.052 (-0.19, 0.29)	66|166467	N

Supplementary Table 4: WHRadjBMI associations in women

Results for any variants set significant in the male-specific, female-specific or sex-combined analysis are shown. Burden analysis was performed using a generalized linear model adjusting for the first 30 PCs of genetic ancestry. The threshold for significance was $P \leq 1.05 \times 10^{-6}$ (Bonferroni correcting for the number of genes and variant masks tested). N = 195,777 women with WHRadjBMI measurements.

**Gene**	**Variant set**	**Gene coordinates**	***P***	**Beta (95% CI) in SD units of WHRadjBMI**	**N (carriers|non-carriers)**	**significant**
PDE3B	pLOF	11:14643691-14874139	6.31E-23	-0.42 (-0.5, -0.33)	555|195222	Y
PLIN1	pLOF	15:89664365-89679417	3.94E-14	-0.39 (-0.49, -0.29)	365|195412	Y
INSR	pLOF	19:7112255-7294405	1.48E-13	-0.88 (-1.1, -0.65)	69|195708	Y
PLIN4	pLOF	19:4502180-4520285	1.68E-12	0.21 (0.16, 0.27)	1070|194707	Y
PLIN4	pLOF+missense	19:4502180-4520285	1.19E-11	0.2 (0.14, 0.25)	1195|194582	Y
PLIN1	pLOF+missense	15:89664365-89679417	1.29E-11	-0.13 (-0.17, -0.095)	2579|193198	Y
ACVR1C	pLOF+missense	2:157526767-157628887	2.25E-08	-0.17 (-0.23, -0.11)	1052|194725	Y
ACVR1C	missense	2:157526767-157628887	2.88E-08	-0.17 (-0.23, -0.11)	1035|194742	Y
ABCA1	pLOF+missense	9:104781002-104928246	4.24E-08	-0.082 (-0.11, -0.053)	4511|191266	Y
INSR	pLOF+missense	19:7112255-7294405	4.56E-08	-0.2 (-0.27, -0.13)	773|195004	Y
COL5A3	pLOF	19:9959561-10010532	8.73E-08	0.33 (0.21, 0.45)	259|195518	Y
SLC35F5	missense	2:113702677-113756823	1.78E-07	-0.095 (-0.13, -0.06)	3002|192775	Y
KEAP1	pLOF+missense	19:10486120-10503378	3.41E-07	0.29 (0.18, 0.4)	307|195470	Y
SLC35F5	pLOF+missense	2:113702677-113756823	3.98E-07	-0.089 (-0.12, -0.055)	3256|192521	Y
KEAP1	missense	19:10486120-10503378	5.40E-07	0.3 (0.18, 0.41)	280|195497	Y
ABCA1	missense	9:104781002-104928246	5.93E-07	-0.077 (-0.11, -0.046)	4289|191488	Y
INHBE	pLOF+missense	12:57455291-57458013	2.19E-06	-0.21 (-0.3, -0.12)	503|195274	N
TRIM40	pLOF+missense	6:30135998-30148773	5.49E-06	0.092 (0.053, 0.13)	2409|193368	N
INHBE	pLOF	12:57455291-57458013	3.06E-05	-0.22 (-0.33, -0.12)	340|195437	N

SLC5A3	missense	21:34073523-34106262	5.83E-05	0.07 (0.036, 0.10)	3331|192446	N
PYGM	pLOF	11:64746389-64760715	4.57E-04	0.081 (0.036, 0.13)	1857|193920	N
ANKRD12	pLOF	18:9136753-9285985	0.002	0.27 (0.1, 0.44)	130|195647	N
LIPE	pLOF+missense	19:42401507-42427426	0.04	0.033 (0.0023, 0.064)	4084|191693	N
LIPE	missense	19:42401507-42427426	0.09	0.027 (-0.0041, 0.059)	3895|191882	N
GIGYF1	pLOF	7:100679506-100694280	0.78	-0.037 (-0.3, 0.23)	54|195723	N

Supplementary Table 5: Meta-analysis of WHRadjBMI in men and women

Gene burden results from sex-stratified analysis of WHRadjBMI were meta-analyzed using an inverse-variance weighted method implemented in METAL. Results for any variant set significant in the male-specific, female-specific or sex-combined analysis are shown. P_{het}; heterogeneity P-value from Cochran's Q test ($P \leq 0.05$ suggests evidence of heterogeneity).

Gene	Variant set	*P*	Beta (95% CI) in SD units of WHRadjBMI	Direction	P_{het}	sig	het
PLIN1	pLOF	1.18E-21	-0.36 (-0.44, -0.29)	--	0.41	Y	N
PDE3B	pLOF	2.61E-16	-0.26 (-0.32, -0.19)	--	1.58E-08	Y	Y
PLIN1	pLOF+missense	1.15E-14	-0.11 (-0.14, -0.084)	--	0.12	Y	N
ACVR1C	missense	1.21E-09	-0.14 (-0.18, -0.094)	--	0.12	Y	N
ACVR1C	pLOF+missense	1.49E-09	-0.14 (-0.18, -0.093)	--	0.10	Y	N
SLC5A3	missense	1.06E-08	0.073 (0.048, 0.098)	++	0.77	Y	N
PYGM	pLOF	1.35E-08	0.098 (0.064, 0.13)	++	0.28	Y	N
INHBE	pLOF+missense	1.36E-08	-0.19 (-0.25, -0.12)	--	0.44	Y	N
PLIN4	pLOF+missense	2.19E-08	0.12 (0.077, 0.16)	++	8.53E-05	Y	Y
PLIN4	pLOF	2.62E-08	0.13 (0.081, 0.17)	++	1.22E-05	Y	Y
INHBE	pLOF	4.44E-08	-0.22 (-0.30, -0.14)	--	0.88	Y	N
KEAP1	pLOF+missense	7.95E-08	0.23 (0.15, 0.32)	++	0.13	Y	N
ANKRD12	pLOF	1.27E-07	0.32 (0.2, 0.45)	++	0.38	Y	N
KEAP1	missense	1.77E-07	0.24 (0.15, 0.33)	++	0.12	Y	N
INSR	pLOF	6.12E-07	-0.43 (-0.59, -0.26)	-+	4.5E-08	Y	Y
COL5A3	pLOF	1.1E-06	0.22 (0.13, 0.31)	++	0.009	N	Y
TRIM40	pLOF+missense	1.84E-06	0.072 (0.042, 0.1)	++	0.13	N	N
ABCA1	pLOF+missense	7.00E-06	-0.05 (-0.071, -0.028)	--	0.001	N	Y
INSR	pLOF+missense	1.32E-05	-0.11 (-0.17, -0.063)	--	8.29E-04	N	Y
ABCA1	missense	3.45E-05	-0.047 (-0.069, -0.025)	--	0.004	N	Y
GIGYF1	pLOF	2.96E-04	0.32 (0.14, 0.49)	-+	6.38E-04	N	Y
SLC35F5	missense	0.003	-0.04 (-0.066, -0.013)	-+	4.93E-06	N	Y
SLC35F5	pLOF+missense	0.005	-0.037 (-0.062, -0.011)	-+	7.6E-06	N	Y
LIPE	missense	0.02	-0.027 (-0.051, -0.0043)	+-	5.17E-07	N	Y
LIPE	pLOF+missense	0.04	-0.024 (-0.046, -0.0011)	+-	1.07E-07	N	Y

Supplementary Table 6: Replication of gene-level associations in AMP-T2D-GENES

Gene-based analysis of WHRadjBMI was performed using a burden test with linear regression in up to 27,380 unrelated individuals. The analysis used 10 PCs of genetic ancestry, sample cohort subgroup, and sequencing technology as covariates. The threshold for statistical significance adjusting for 10 genes tested is $P \leq 0.005$ with $P \leq 0.05$ indicating nominal significance.

**Gene**	**Variant set**	***P***	**Beta (95% CI)**	**N**	**N carriers**
INHBE	pLOF	9.41×10^{-4}	-1.03 (-1.60, -0.42)	13456	10
INHBE	pLOF+missense	0.04	-0.31 (-0.61, -0.0076)	20052	41
PLIN1	pLOF+missense	0.05	-0.13 (-0.27, 0.002)	25380	204
PLIN4	pLOF	0.11	0.15 (-0.032, 0.32)	25761	121
PLIN1	pLOF	0.17	-0.26 (-0.64, 0.12)	19549	26
PDE3B	pLOF	0.18	-0.32 (-0.78, 0.14)	13270	17
KEAP1	missense	0.20	0.28 (-0.14, 0.70)	19995	21
KEAP1	pLOF+missense	0.20	0.263 (-0.14, 0.66)	19995	23
INSR	pLOF	0.29	-0.33 (-0.95, 0.29)	6269	10
SLC5A3	missense	0.42	0.19 (-0.27, 0.66)	16430	17
ACVR1C	missense	0.50	0.08 (-0.15, 0.31)	25154	70
ACVR1C	pLOF+missense	0.50	0.08 (-0.15, 0.31)	25154	70
PLIN4	pLOF+missense	0.55	0.03 (-0.07, 0.13)	25761	401
COL5A3	pLOF	0.76	0.06 (-0.30, 0.41)	21776	29
PYGM	pLOF	0.85	0.02 (-0.21, 0.26)	24037	67

Supplementary Table 7: Sensitivity analysis in White outgroup of UKB

Association results for genes significant in the discovery analysis with ≥ 5 rare variant carriers in the White outgroup. Burden analysis of WHRadjBMI was performed using a generalized linear model. The direction of effect in the sensitivity analysis and discovery analysis respectively is shown as well as meta-analysis of these results. The threshold for statistical significance adjusting for 11 genes tested is *P* ≤ 0.003 with *P* ≤ 0.05 indicating nominal significance

**Gene**	**Variant set**	***P***	**Beta (95% CI)**	**N carrier**	**Significance** ** *P* ≤ 0.05 * *P* ≤ 0.003	**Direction**	***P* meta**	**Beta meta (95% CI)**
PLIN1	pLOF	2.38E-04	-0.48 (-0.73, -0.22)	59	**	--	3.57E-23	-0.36 (-0.43, -0.29)
PLIN1	pLOF+missense	0.16	-0.061 (-0.14, 0.023)	560		--	3.16E-14	-0.10 (-0.13, -0.077)
PLIN4	pLOF	0.006	0.23 (0.067, 0.39)	149	*	++	6.6E-11	0.14 (0.01, 0.18)
PLIN4	pLOF+missense	0.002	0.24 (0.086, 0.38)	173	**	++	3.93E-11	0.14 (0.095, 0.17)
INSR	pLOF	0.007	-0.96 (-1.6, -0.26)	8	*	--	6.44E-09	-0.48 (-0.64, -0.32)
INHBE	pLOF	0.44	-0.13 (-0.44, 0.19)	38		--	4.24E-08	-0.21 (-0.29, -0.14)
INHBE	pLOF+missense	0.64	-0.063 (-0.33, 0.2)	54		--	2.65E-08	-0.18 (-0.24, -0.11)
ACVR1C	missense	0.58	-0.052 (-0.24, 0.13)	114		--	8.3E-10	-0.14 (-0.18, -0.093)
ACVR1C	pLOF+missense	0.57	-0.052 (-0.23, 0.13)	118		--	9.26E-10	-0.13 (-0.18, -0.092)
COL5A3	pLOF	0.40	0.16 (-0.21, 0.54)	27		++	3.07E-07	0.23 (0.14, 0.31)
KEAP1	missense	4.30E-04	0.40 (0.18, 0.62)	79	**	++	9.18E-11	0.27 (0.19, 0.36)
KEAP1	pLOF+missense	4.99E-04	0.38 (0.16, 0.59)	86	**	++	4.31E-11	0.27 (0.19, 0.35)
PDE3B	pLOF	0.15	-0.16 (-0.38, 0.06)	78		--	1.12E-17	-0.26 (-0.32, -0.20)
PYGM	pLOF	0.66	-0.029 (-0.16, 0.1)	220		+-	3.78E-07	0.085 (0.052, 0.12)
SLC5A3	missense	0.11	0.064 (-0.013, 0.14)	662		++	5.09E-09	0.071 (0.047, 0.095)

Supplementary Table 8: *INHBE* pLOF leave-one-variant-out analysis in UKB

We performed leave-one-variant-out analysis for *INHBE* pLOF variants in UKB. Gene-based burden analysis of WHRadjBMI was performed using a generalized linear model excluding one variant at a time from the analysis. Single variant analysis was performed using a linear model.

Variant set	*P* burden	Beta burden (95% CI)	N carrier burden	rsid	consequence	HGVS annotation	MAF	*P* single variant	Beta single variant (95% CI)
INHBE pLOF all variants	4.98E-08	-0.22 (-0.3, -0.14)	618						
INHBE pLOF 12:57456093:G:C	0.34	-0.10 (-0.32, 0.11)	80	rs150777893	splice acceptor variant	NM_031479.4: c.299-1G>C	0.074%	4.31E-08	-0.23 (-0.32, -0.15)
INHBE pLOF 12:57455835:G:T	2.44E-07	-0.21 (-0.3, -0.13)	569	rs375342858	splice donor variant	NM_031479.4: c.298+1G>T	0.007%	0.08	-0.25 (-0.53, 0.03)
INHBE pLOF 12:57455654:C:T	3.19E-08	-0.22 (-0.3, -0.14)	604	""	stop gained	NP_113667.1: p.Arg40Ter	0.002%	0.92	0.03 (-0.49, 0.55)
INHBE pLOF 12:57455698:GT:G	1.49E-08	-0.23 (-0.31, -0.15)	611	""	frameshift variant	NP_113667.1: p.Leu55CysfsTer3	0.001%		
INHBE pLOF 12:57455588:C:T	8.50E-08	-0.21 (-0.29, -0.14)	614	rs750332159	stop gained	NP_113667.1: p.Arg18Ter	0.0005%		
INHBE pLOF 12:57455553:TC:T	4.49E-08	-0.22 (-0.3, -0.14)	616	""	frameshift variant	NP_113667.1: p.Gln7SerfsTer40	0.0003%		
INHBE pLOF 12:57455645:C:T	3.82E-08	-0.22 (-0.3, -0.14)	616	""	stop gained	NP_113667.1: p.Gln37Ter	0.0003%		
INHBE pLOF 12:57455742:A:G	4.01E-08	-0.22 (-0.3, -0.14)	616	""	splice acceptor variant	NP_113667.1: p.Gln69Arg	0.0003%		

Supplementary Table 9: *INHBE* pLOF leave-one-variant-out analysis in AMP-T2D-GENES

We performed leave-one-variant-out analysis for *INHBE* pLOF in AMP-T2D-GENES. Analysis was performed using a burden test with linear regression excluding one variant at a time from the analysis.

Variant set	*P*	Beta in SD (95% CI)	N	N carrier	rsid	consequence
INHBE pLOF all variants	9.41E-04	-1.03 (-1.64, -0.42)	13456	10		
INHBE pLOF exclude 12:57850337 C/T,A	0.03	-0.77 (-1.45, -0.09)	12550	8	rs146517777	stop gain (Tyr253Ter)
INHBE pLOF exclude 12:57849876 G/C	0.008	-1.16 (-2.01, -0.31)	7100	5	rs150777893	splice acceptor variant
INHBE pLOF exclude 12:57849618 G/T	1.18E-04	-1.26 (-1.90, -0.62)	13456	9	rs375342858	splice donor variant
INHBE pLOF exclude 12:57850164 G/T	0.006	-0.95 (-1.63, -0.27)	11813	8	rs764817559	stop gain (Glu196Ter)

Supplementary Table 10: Association of WHRadjBMI genes with unadjusted WHR and BMI.

For genes associated with WHRadjBMI, the effects on WHR (without BMI adjustment) and BMI are shown. Analysis was performed using a generalized linear model adjusting for age, sex, and the first 30 PCs of genetic ancestry. Whether variant sets are significant correcting for the number of variant sets and phenotypes tested is indicated ($P < 0.0016$).

**Title**	**Variant set**	***P***	**Beta in SD (95% CI)**	**significant**
WHR	*ACVR1C* missense	1.24E-05	-0.076 (-0.11, -0.042)	Y
BMI	*ACVR1C* missense	0.16	0.031 (-0.013, 0.076)	N
WHR	*ACVR1C* pLOF+missense	2.77E-05	-0.072 (-0.11, -0.038)	Y
BMI	*ACVR1C* pLOF+missense	0.09	0.038 (-0.0056, 0.082)	N
WHR	*ANKRD12* pLOF	3.15E-09	0.27 (0.18, 0.37)	Y
BMI	*ANKRD12* pLOF	0.005	0.17 (0.052, 0.29)	N
WHR	*COL5A3* pLOF	2.26E-06	0.16 (0.095, 0.23)	Y
BMI	*COL5A3* pLOF	0.57	0.025 (-0.063, 0.11)	N
WHR	*INHBE* pLOF	3.57E-05	-0.12 (-0.18, -0.066)	Y
BMI	*INHBE* pLOF	0.77	0.011 (-0.066, 0.089)	N
WHR	*INHBE* pLOF+missense	2.95E-04	-0.09 (-0.14, -0.041)	Y
BMI	*INHBE* pLOF+missense	0.13	0.049 (-0.015, 0.11)	N
WHR	*INSR* pLOF	8.53E-07	-0.32 (-0.45, -0.19)	Y
BMI	*INSR* pLOF	0.64	0.039 (-0.13, 0.2)	N
WHR	*KEAP1* missense	7.31E-06	0.15 (0.087, 0.22)	Y
BMI	*KEAP1* missense	0.69	-0.018 (-0.11, 0.07)	N
WHR	*KEAP1* pLOF+missense	1.01E-05	0.14 (0.08, 0.21)	Y
BMI	*KEAP1* pLOF+missense	0.59	-0.023 (-0.11, 0.061)	N
WHR	*PDE3B* pLOF	3.36E-07	-0.12 (-0.17, -0.074)	Y
BMI	*PDE3B* pLOF	1.41E-04	0.12 (0.057, 0.18)	Y
WHR	*PLIN1* pLOF	1.07E-11	-0.2 (-0.25, -0.14)	Y
BMI	*PLIN1* pLOF	0.08	0.066 (-0.0079, 0.14)	N
WHR	*PLIN1* pLOF+missense	2.07E-05	-0.046 (-0.068, -0.025)	Y
BMI	*PLIN1* pLOF+missense	1.67E-04	0.054 (0.026, 0.082)	Y
WHR	*PLIN4* pLOF	2.06E-04	0.063 (0.03, 0.096)	Y
BMI	*PLIN4* pLOF	0.010	-0.057 (-0.1, -0.014)	N
WHR	*PLIN4* pLOF+missense	1.99E-04	0.059 (0.028, 0.091)	Y
BMI	*PLIN4* pLOF+missense	0.008	-0.055 (-0.096, -0.014)	N
WHR	*PYGM* pLOF	3.88E-05	0.053 (0.028, 0.079)	Y
BMI	*PYGM* pLOF	0.61	-0.0086 (-0.042, 0.025)	N
WHR	*SLC5A3* missense	7.39E-09	0.055 (0.037, 0.074)	Y
BMI	*SLC5A3* missense	0.02	0.029 (0.0048, 0.054)	N

Supplementary Table 11: Association of WHRadjBMI genes with metabolic syndrome score

The association of WHRadjBMI-associated genes with MetS score was tested using ordinal regression adjusting for age, sex, and the first 30 PCs of genetic ancestry. The p-value and beta from the ordinal regression are shown as well as the change in MetS score expressed in terms of number of MetS traits.

**Title**	**Variant set**	***P***	**Beta from ordinal regression (95% CI)**	**Change in MetS score (95% CI)**	**N carrier measured**
MetS score	*PLIN1* pLOF	1.36E-08	-0.44 (-0.59, -0.29)	-0.31 (-0.41, -0.21)	537
MetS score	*PLIN1* pLOF+missense	9.69E-04	-0.097 (-0.15, -0.039)	-0.074 (-0.11, -0.036)	3691
MetS score	*PDE3B* pLOF	5.82E-08	-0.34 (-0.47, -0.22)	-0.22 (-0.3, -0.14)	811
MetS score	*ACVR1C* missense	0.004	-0.14 (-0.23, -0.046)	-0.085 (-0.15, -0.024)	1448
MetS score	*ACVR1C* pLOF+missense	0.006	-0.13 (-0.22, -0.036)	-0.08 (-0.14, -0.019)	1468
MetS score	*INHBE* pLOF	0.02	-0.19 (-0.35, -0.03)	-0.12 (-0.23, -0.016)	483
MetS score	*INHBE* pLOF+missense	0.04	-0.14 (-0.27, -0.0078)	-0.08 (-0.17, 0.0057)	727
MetS score	*PYGM* pLOF	0.006	0.097 (0.028, 0.17)	0.067 (0.021, 0.11)	2577
MetS score	*ANKRD12* pLOF	0.03	0.28 (0.033, 0.53)	0.2 (0.045, 0.36)	215
MetS score	*INSR* pLOF	0.04	-0.35 (-0.68, -0.013)	-0.22 (-0.44, -0.0011)	112
MetS score	*KEAP1* pLOF+missense	0.22	0.11 (-0.065, 0.29)	0.08 (-0.034, 0.19)	409
MetS score	*KEAP1* missense	0.25	0.11 (-0.075, 0.29)	0.085 (-0.034, 0.2)	380
MetS score	*COL5A3* pLOF	0.37	0.083 (-0.098, 0.26)	0.06 (-0.06, 0.18)	378
MetS score	*PLIN4* pLOF	0.45	0.035 (-0.055, 0.12)	0.015 (-0.044, 0.075)	1518
MetS score	*PLIN4* pLOF+missense	0.46	0.032 (-0.053, 0.12)	0.015 (-0.041, 0.071)	1717
MetS score	*SLC5A3* missense	0.62	0.013 (-0.038, 0.064)	0.0079 (-0.026, 0.041)	4831

Supplementary Table 12: Association of *INHBE* pLOF with metabolic traits

The association of *INHBE* pLOF with various metabolic traits was performed using a generalized linear model adjusting for 30 PCs and other covariates as indicated in the Methods. The effect in standard deviations (SD) and clinical units is shown. VATadjBMI, visceral adipose tissue adjusted for BMI; ASATadjBMI, abdominal subcutaneous adipose tissue adjusted for BMI.

Title	*P*	Beta in SD (95% CI)	Beta in clinical units (95% CI)	N carrier measured	Clinical units
WHRadjBMI	4.98E-08	-0.22 (-0.3, -0.14)		618	
WHR	3.57E-05	-0.12 (-0.18, -0.07)	-0.01 (-0.02, -0.006)	619	
Hip circumference	0.37	0.035 (-0.04, 0.11)	0.33 (-0.39, 1.00)	619	cm
Waist circumference	0.08	-0.06 (-0.13, 0.01)	-0.82 (-1.8, 0.11)	619	cm
BMI	0.58	0.02 (-0.06, 0.10)	0.11 (-0.27, 0.48)	596	kg/m2
Triglycerides	9.65E-04	-0.13 (-0.21, -0.05)	-0.13 (-0.21, -0.06)	594	mmol/L
HDL cholesterol	0.01	0.10 (0.02, 0.18)	0.04 (0.01, 0.07)	550	mmol/L
LDL cholesterol	0.26	-0.05 (-0.13, 0.03)	-0.04 (-0.11, 0.03)	594	mmol/L
LDL adj medication	0.11	-0.06 (-0.14, 0.02)	-0.054 (-0.12, 0.01)	594	mmol/L
ApoB	0.06	-0.076 (-0.16, 0.004)	-0.02 (-0.04, 0.001)	593	g/L
ALT	0.04	-0.08 (-0.15, -0.004)	-1.10 (-2.20, -0.06)	595	U/L
HbA1c	0.78	-0.011 (-0.087, 0.065)	-0.074 (-0.59, 0.44)	595	mmol/mol
Glucose	0.21	0.05 (-0.03, 0.13)	0.07 (-0.04, 0.17)	551	mmol/L
Fasting glucose (GP)	0.03	-0.17 (-0.33, -0.02)	-0.37 (-0.70, -0.03)	90	mmol/L
Diastolic BP	0.10	-0.07 (-0.15, 0.01)	-0.71 (-1.60, 0.15)	574	mmHg
Systolic BP	0.46	-0.03 (-0.10, 0.05)	-0.56 (-2.10, 0.94)	574	mmHg
ASATadjBMI	0.12	-0.27 (-0.61, 0.07)		33	
VATadjBMI	0.26	-0.20 (-0.54, 0.14)		33	
Liver fat	0.93	-0.02 (-0.46, 0.42)	-0.09 (-2.10, 1.90)	18	%

Supplementary Table 13: Association of *INHBE* pLOF with cardiometabolic disease

The association of *INHBE* pLOF with disease diagnosis was tested using a mixed-effects model with the effect on disease risk from this regression shown as an odds ratio ("OR"). The odds ratio predicted from a Mendelian Randomization study of WHRadjBMI [1] is also shown ("MR-predicted OR") along with the total number of individuals we would need to sequence to detect a statistically significant effect on disease risk ("N total for *P* = 0.05").

**Diagnosis**	***P***	**OR (95% CI)**	**N cases| N controls**	**N carrier cases**	**MAF**	**MR-predicted OR**	**N total for *P* = 0.05**
Coronary Heart Disease (phecode 411)	0.05	0.78 (0.60, 1.00)	48193|350357	70	0.08%	0.92	7,357,216
Type 2 diabetes (phecode 250.2)	0.65	0.94 (0.70, 1.24)	32383|366167	51	0.08%	0.88	4,925,516

Supplementary Table 14: Association of rs150777893 with INHBC protein levels in an Icelandic plasma proteomic study

The *INHBE* pLOF variant rs150777893 was examined in a recent independent study of plasma proteins performed in the Icelandic population [2]. The association with circulating INHBC levels is shown.

**Title**	**rsid**	**gene**	***P***	**Beta in SD (95% CI)**
INHBC protein	rs150777893	*INHBE*	0.006	0.52 (0.15, 0.90)

Supplementary Table 15: CNVs deleting *INHBE* in WES and WGS data from UKB

CNVs impacting *INHBE* that are present in the UKB WES data (n=454,756 participants) and/or WGS data (n=150,119 participants). Two of the CNVs identified in the WES data were also identified using the WGS data, with slightly different breakpoints as shown. The start and end positions for these deletions in hg38 coordinates are provided and the genes overlapped are indicated. *INHBE* hg38 coordinates used were 12:57455291-57458013.

**CNV Chr:Start-End (WES)**	**CNV Type (WES)**	**Genes overlapped (WES)**	**CNV Chr:Start-End (WGS)**	**CNV Type (WGS)**	**Genes overlapped (WGS)**
12:57,449,276-57,490,644	DEL	*INHBE, INHBC, ARHGAP9, GLI1, MARS1*	12:57,438,169-57,495,228	DEL	*INHBE, INHBC, GLI1, ARHGAP9, MARS1*
12:57,449,276-57,456,848	DEL	*INHBC, INHBE*	NA (individual has no WGS data)		
12:57,418,185-57,456,848	DEL	*INHBC, INHBE, R3HDM2*	12:57,414,374-57,458,676	DEL	*INHBE, INHBC, R3HDM2*
			12:57,456,367-57,458,350	DEL	*INHBE*
			12:57,456,367 -57,458,350	DEL	*INHBE*

Supplementary Table 16: PheWAS of *ACVR1C* damaging missense variants

Phenome-wide significant associations of *ACVR1C* damaging missense variants ($P \leq 5x10^{-5}$) are shown along with associations with selected cardiometabolic traits and diseases. Association testing was performed using a generalized linear model for quantitative traits and a mixed-effects model for disease diagnoses.

**Title**	**Variant set**	***P***	**Beta in SD or OR (95% CI)**	**N carrier measured/ N carrier cases**	**phenome-wide significant**
WHRadjBMI	*ACVR1C* missense	6.24E-10	-0.14 (-0.19, -0.097)	1892	Y
Birth weight	*ACVR1C* missense	1.92E-23	0.30 (0.24, 0.36)	1088	Y
WHR	*ACVR1C* missense	1.24E-05	-0.076 (-0.11, -0.042)	1894	Y
Whole body water mass	*ACVR1C* missense	6.96E-04	0.048 (0.02, 0.077)	1857	N
BMI	*ACVR1C* missense	0.17	0.031 (-0.013, 0.076)	1857	N
Waist circumference	*ACVR1C* missense	0.67	-0.009 (-0.048, 0.031)	1894	N
Triglycerides	*ACVR1C* missense	0.06	-0.043 (-0.088, 0.0014)	1795	N
HDL cholesterol	*ACVR1C* missense	0.08	0.039 (-0.005, 0.082)	1645	N
LDL cholesterol	*ACVR1C* missense	0.26	0.027 (-0.02, 0.073)	1792	N
ApoB	*ACVR1C* missense	0.63	0.011 (-0.035, 0.058)	1791	N
LDL adj medication	*ACVR1C* missense	0.59	0.013 (-0.033, 0.058)	1792	N
ALT	*ACVR1C* missense	0.67	-0.009 (-0.053, 0.034)	1797	N
HbA1c	*ACVR1C* missense	0.06	-0.042 (-0.086, 0.0011)	1806	N
Diastolic BP	*ACVR1C* missense	0.01	-0.06 (-0.11, -0.014)	1754	N
Systolic BP	*ACVR1C* missense	0.04	-0.046 (-0.089, -0.0021)	1754	N
VATadjBMI	*ACVR1C* missense	0.02	-0.23 (-0.42, -0.04)	107	N
ASATadjBMI	*ACVR1C* missense	0.84	-0.02 (-0.21, 0.17)	107	N
Type 2 diabetes	*ACVR1C* missense	0.005	0.78 (0.66, 0.94)	138	N
Coronary Heart Disease	*ACVR1C* missense	0.53	1.0 (0.91, 1.2)	268	N

Supplementary Table 17: PheWAS of *PLIN1* pLOF

Phenome-wide significant associations of *PLIN1* pLOF variants ($P \leq 5x10^{-5}$) are shown along with associations with selected cardiometabolic traits and diseases. Association testing was performed using a generalized linear model for quantitative traits and a mixed-effects model for disease diagnoses.

**Title**	**Variant set**	***P***	**Beta in SD or OR (95% CI)**	**N carrier measured/N carrier cases**	**Phenome-wide significant**
WHRadjBMI	*PLIN1* pLOF	2.12E-20	-0.35 (-0.43, -0.28)	681	Y
HDL cholesterol	*PLIN1* pLOF	3.29E-20	0.34 (0.27, 0.41)	601	Y
ApoA	*PLIN1* pLOF	4.71E-13	0.27 (0.20, 0.34)	594	Y
Triglycerides	*PLIN1* pLOF	9.56E-12	-0.26 (-0.33, -0.18)	651	Y
WHR	*PLIN1* pLOF	1.07E-11	-0.2 (-0.25, -0.14)	682	Y
Reticulocyte count	*PLIN1* pLOF	4.11E-08	-0.21 (-0.29, -0.14)	656	Y
Reticulocyte percentage	*PLIN1* pLOF	5.31E-07	-0.20 (-0.27, -0.12)	656	Y
Hip circumference	*PLIN1* pLOF	3.34E-06	0.18 (0.10, 0.25)	682	Y
HLS reticulocyte count	*PLIN1* pLOF	4.65E-06	-0.18 (-0.25, -0.1)	656	Y
Leukocyte count	*PLIN1* pLOF	9.54E-06	-0.17 (-0.25, -0.095)	665	Y
HLS reticulocyte percentage	*PLIN1* pLOF	4.52E-05	-0.16 (-0.24, -0.083)	656	Y
ApoB	*PLIN1* pLOF	6.77E-04	-0.13 (-0.21, -0.056)	649	N
LDL adj medication	*PLIN1* pLOF	0.004	-0.11 (-0.19, -0.035)	652	N
Diastolic BP	*PLIN1* pLOF	0.02	-0.08 (-0.15, -0.01)	636	N
Systolic BP	*PLIN1* pLOF	0.03	-0.08 (-0.16, -0.007)	636	N
HbA1c	*PLIN1* pLOF	0.08	-0.065 (-0.14, 0.007)	656	N
BMI	*PLIN1* pLOF	0.08	0.067 (-0.008, 0.14)	669	N
LDL cholesterol	*PLIN1* pLOF	0.09	-0.066 (-0.14, 0.011)	652	N
Waist circumference	*PLIN1* pLOF	0.34	-0.032 (-0.098, 0.034)	682	N
ASATadjBMI	*PLIN1* pLOF	0.21	0.21 (-0.12, 0.54)	36	N
VATadjBMI	*PLIN1* pLOF	0.88	-0.025 (-0.35, 0.3)	36	N
Type 2 diabetes	*PLIN1* pLOF	0.11	0.81 (0.62, 1.05)	52	N
Coronary Heart Disease	*PLIN1* pLOF	0.07	0.81 (0.65, 1.02)	79	N
Essential hypertension	*PLIN1* pLOF	0.53	0.95 (0.8, 1.12)	241	N

AMP-T2D-GENES Consortium members

Carlos A. Aguilar-Salinas[1], Gil Atzmon[2,3,4], Francisco Barajas-Olmos[5], Nir Barzilai[2,4], John Blangero[6], Eric Boerwinkle[7,8], Lori L. Bonnycastle[9], Erwin Bottinger[10], Donald W Bowden[11,12,13], Federico Centeno- Cruz[5], John C. Chambers[14,15], Edmund Chan[16], Juliana Chan[17,18,19,20], Ching-Yu Cheng[21,22,23], Yoon Shin Cho[24], Cecilia Contreras-Cubas[5], Emilio Córdova[5], Adolfo Correa[25], Ralph A. DeFronzo[26], Ravindranath Duggirala[6], Josée Dupuis[27], Humberto García-Ortiz[5], Christian Gieger[28,29,30], Benjamin Glaser[31], Clicerio Gonzalez[32], Ma Elena Gonzalez[33], Niels Grarup[34], Leif Groop[35,36], Myron Gross[37], Christopher Haiman[38], Sohee Han[39], Craig L. Hanis[7], Torben Hansen[34], Nancy L. Heard-Costa[40,41], Brian E. Henderson[38], Juan Manuel Malacara Hernandez[42], Mi Yeong Hwang[39], Sergio Islas-Andrade[5], Marit E. Jørgensen[43,44,45], Hyun Min Kang[46], Bong-Jo Kim[39], Young Jin Kim[39], Heikki A. Koistinen[47,48,49], Jaspal Singh Kooner[50,51,52], Johanna Kuusisto[53], Soo Heon Kwak[54], Markku Laakso[53], Leslie A. Lange[55], Juyoung Lee[39], Jong-Young Lee[56], Donna M. Lehman[26], Allan Linneberg[57,58,59], Jianjun Liu[16,60,61], Ruth Loos[10,62], Valeriya Lyssenko[33,63], Ronald C.W. Ma[17,18,19,20], Angélica Martínez-Hernández[5], James B. Meigs[64,65,66], Thomas Meitinger[67,68], Elvia Mendoza-Caamal[5], Karen L. Mohlke[69], Andrew D. Morris[70,71], Alanna C. Morrison[7,72], Maggie C.Y. Ng[11,12,13], Peter Nilsson[73], Christopher J. O'Donnell[74,75,76,77], Lorena Orozco[5], Colin N.A. Palmer[78], Kyong Soo Park[54,79,80], Oluf Pedersen[34], Wendy S. Post[81], Michael Preuss[10], Bruce M. Psaty[82,83], Ramachandran S. Vasan[40,84], Alexander P. Reiner[85], Cristina Revilla-Monsalve[5], Stephen S. Rich[86], Jerome I. Rotter[87], Zhe Wang[10], Maria Eugenia Garay Sevilla[88,89], Xueling Sim[60], Rob Sladek[90,91,92], Kerrin S. Small[93], Wing Yee So[17,18,19], Timothy D. Spector[93], Konstantin Strauch[29,94], Tim M. Strom[67,95], E-Shyong Tai[16,60,96], Claudia H.T. Tam[17,18,19], Yik Ying Teo[60,96], Farook Thameem[97], Brain Tomlinson[98], Russell P. Tracy[99,100], Tiinamaija Tuomi[101,102], Jaakko Tuomilehto[103,104,105,106], Teresa Tusié-Luna[107,108], Rob M. van Dam[16,60,109], James G. Wilson[110], Daniel R. Witte[111,112], Tien-Yin Wong[21,22,23], Peter Dornbos[64,74,113], Jason A. Flannick[64,74,113], Jose C. Florez[64,65,114], Mark I. McCarthy[115], Michael Boehnke[46], Noël P. Burtt[64]

Affiliations:

[1]Instituto Nacional de Ciencias Medicas y Nutricion, Mexico City, Mexico

[2]Department of Medicine, Albert Einstein College of Medicine, New York, NY, USA. [3]Faculty of Natural Science, University of Haifa, Haifa, Israel.

[4]Department of Genetics, Albert Einstein College of Medicine, New York, NY, USA

[5]Instituto Nacional de Medicina Genómica, Mexico City, Mexico

[6]Department of Human Genetics and South Texas Diabetes and Obesity Institute, University of Texas Rio Grande Valley, Brownsville and Edinburg, TX, USA

[7]Human Genetics Center, School of Public Health, The University of Texas Health Science Center at Houston, Houston, TX, USA

[8]Human Genome Sequencing Center, Baylor College of Medicine, Houston, TX, USA.

[9]Medical Genomics and Metabolic Genetics Branch, National Human Genome Research Institute, National Institutes of Health, Bethesda, MD, USA

[10]The Charles Bronfman Institute for Personalized Medicine, Icahn School of Medicine at Mount Sinai, New York, NY, USA

[11]Center for Diabetes Research, Wake Forest School of Medicine, Winston-Salem, NC, USA.

[12]Center for Genomics and Personalized Medicine Research, Wake Forest School of Medicine, Winston-Salem, NC, USA.

[13]Department of Biochemistry, Wake Forest School of Medicine, Winston-Salem, NC, USA.

[14]Department of Epidemiology and Biostatistics, Imperial College London, London, UK.

[15]Lee Kong Chian School of Medicine, Nanyang Technological University, Singapore, Singapore.

[16]Department of Medicine, Yong Loo Lin School of Medicine, National University of Singapore and National University Health System, Singapore, Singapore.

[17]Department of Medicine and Therapeutics, The Chinese University of Hong Kong, Hong Kong, China.

[18]Chinese University of Hong Kong-Shanghai Jiao Tong University Joint Research Centre in Diabetes Genomics and Precision Medicine, The Chinese University of Hong Kong, Hong Kong, China.

[19]Hong Kong Institute of Diabetes and Obesity, The Chinese University of Hong Kong, Hong Kong, China.

[20]Li Ka Shing Institute of Health Sciences, The Chinese University of Hong Kong, Hong Kong, China.

[21]Singapore Eye Research Institute, Singapore National Eye Centre, Singapore, Singapore

[22]Ophthalmology & Visual Sciences Academic Clinical Program (Eye ACP), Duke-NUS Medical School, Singapore, Singapore

[23]Department of Ophthalmology, Yong Loo Lin School of Medicine, National University of Singapore and National University Health System, Singapore, Singapore.

[24]Department of Biomedical Science, Hallym University, Chuncheon, South Korea

[25]Department of Medicine, University of Mississippi Medical Center, Jackson, MS, USA.

[26]Department of Medicine, University of Texas Health San Antonio (aka University of Texas Health Science Center at San Antonio), San Antonio, TX, USA

[27]Department of Biostatistics, Boston University School of Public Health, Boston, MA, USA.

[28]Research Unit Molecular Epidemiology, Helmholtz Zentrum München, German Research Center for Environmental Health, Neuherberg, Germany.

[29]Institute of Epidemiology, Helmholtz Zentrum München, German Research Center for Environmental Health, Neuherberg, Germany.

[30]German Center for Diabetes Research (DZD), Neuherberg, Germany.

[31]Endocrinology and Metabolism Service, Hadassah-Hebrew University Medical Center, Jerusalem, Israel.

[32]Unidad de Investigacion en Diabetes y Riesgo Cardiovascular, Instituto Nacional de Salud Publica, Cuernavaca, Mexico.

[33]Centro de Estudios en Diabetes, Mexico City, Mexico.

[34]Novo Nordisk Foundation Center for Basic Metabolic Research, Faculty of Health and Medical Sciences, University of Copenhagen, Copenhagen, Denmark.

[35]Department of Clinical Sciences, Diabetes and Endocrinology, Lund University Diabetes Centre, Malmö, Sweden.

[36]Institute for Molecular Medicine Finland, University of Helsinki, Helsinki, Finland.

[37]Department of Laboratory Medicine and Pathology, University of Minnesota, Minneapolis, MN, USA.

[38]Department of Preventive Medicine, Keck School of Medicine of USC, Los Angeles, CA, USA.

[39]Division of Genome Research, Center for Genome Science, National Institute of Health, Chungcheongbuk-do, South Korea.

[40]Boston University and National Heart Lung and Blood Institute's Framingham Heart Study, Framingham, MA, USA.

[41]Department of Neurology, Boston University School of Medicine, Boston, MA, USA.

[42]Department of Medical Science, División of Health Science. University of Guanjuato. Campus León. León, Gto. México.

[43]Steno Diabetes Center Copenhagen, Gentofte, Denmark.

[44]National Institute of Public Health, University of Southern Denmark, Copenhagen, Denmark.

[45]Greenland Centre for Health Research, University of Greenland, Nuuk, Greenland.

[46]Department of Biostatistics and Center for Statistical Genetics, University of Michigan, Ann Arbor, MI, USA.

[47]Department of Public Health Solutions, Finnish Institute for Health and Welfare, Helsinki, Finland.

[48]University of Helsinki and Department of Medicine, Helsinki University Central Hospital, Helsinki, Finland.

[49]Minerva Foundation Institute for Medical Research, Helsinki, Finland.

[50]Department of Cardiology, Ealing Hospital, London North West Healthcare NHS Trust, London, UK.

[51]MRC-PHE Centre for Environment and Health, Imperial College London, London, UK. Imperial College Healthcare NHS Trust, Imperial College London, London, UK. [52]National Heart and Lung Institute, Imperial College London, London, UK.

[53]Institute of Clinical Medicine, Internal Medicine, University of Eastern Finland and Kuopio University Hospital, Kuopio, Finland.

[54]Department of Internal Medicine, Seoul National University Hospital, Seoul, South Korea.

[55]Department of Medicine, University of Colorado Denver, Anschutz Medical Campus, Aurora, CO, USA.

[56]Oneomics Soonchunhyang Mirae Medical Center, Bucheon-si Gyeonggi-do, Republic of Korea.

[57]Department of Clinical Medicine, Faculty of Health and Medical Sciences, University of Copenhagen, Copenhagen, Denmark.

[58]Center for Clinical Research and Prevention, Bispebjerg and Frederiksberg Hospital, Copenhagen, Denmark.

[59]Department of Clinical Experimental Research, Rigshospitalet, Copenhagen, Denmark.

[60]Saw Swee Hock School of Public Health, National University of Singapore and National University Health System, Singapore, Singapore.

[61]Genome Institute of Singapore, Agency for Science Technology and Research, Singapore, Singapore.

[62]The Mindich Child Health and Development Institute, Ichan School of Medicine at Mount Sinai, New York, NY, USA.

[63]Department of Clinical Science, University of Bergen, Bergen, Norway.

[64]Program in Medical and Population Genetics, Broad Institute of MIT and Harvard, Cambridge, MA, USA.

[65]Department of Medicine, Harvard Medical School, Boston, MA, USA.

[66]Division of General Internal Medicine, Massachusetts General Hospital, Boston, MA, USA.

[67]Institute of Human Genetics, Technical University of Munich, Munich, Germany.

[68]German Centre for Cardiovascular Research (DZHK), Partner Site Munich Heart Alliance, Munich, Germany.

[69]Department of Genetics, University of North Carolina Chapel Hill, Chapel Hill, NC, USA.

[70]Wellcome Centre for Human Genetics, Nuffield Department of Medicine, University of Oxford, Oxford, UK.

[71]Department of Biostatistics, University of Liverpool, Liverpool, UK.

[72]Department of Epidemiology, Human Genetics, and Environmental Sciences, School of Public Health, The University of Texas Health Science Center at Houston, Houston, TX, USA.

[73]Department of Clinical Sciences, Medicine, Lund University, Malmö, Sweden.

[74]Department of Pediatrics, Harvard Medical School, Boston, MA, USA.

[75]Section of Cardiology, Department of Medicine, VA Boston Healthcare, Boston, MA, USA.

[76]Brigham and Women’s, Hospital, Boston, MA, USA.

[77]Intramural Administration Management Branch, National Heart Lung and Blood Institute, NIH, Framingham, MA, USA.

[78]Pat Macpherson Centre for Pharmacogenetics and Pharmacogenomics, University of Dundee, Dundee, UK

[79]Department of Internal Medicine, Seoul National University College of Medicine, Seoul, South Korea.

[80]Department of Molecular Medicine and Biopharmaceutical Sciences, Graduate School of Convergence Science and Technology, Seoul National University, Seoul, South Korea.

[81]Division of Cardiology, Department of Medicine, Johns Hopkins University, Baltimore, MD, USA.

[82]Cardiovascular Health Research Unit, Departments of Medicine, Epidemiology, and Health Services, University of Washington, Seattle, WA, USA.

[83]Kaiser Permanente Washington Research Institute, Seattle, WA, USA.

[84]Preventive Medicine & Epidemiology, and Cardiovascular Medicine, Medicine, Boston University School of Medicine, and Epidemiology, Boston University School of Public health, Boston, MA, USA.

[85]Fred Hutchinson Cancer Research Center, Seattle, WA, USA.

[86]Center for Public Health Genomics, University of Virginia School of Medicine, Charlottesville, VA, USA

[87]The Institute for Translational Genomics and Population Sciences, Department of Pediatrics, The Lundquist Institute for Biomedical Innovation (formerly Los Angeles Biomedical Research Institute) at Harbor-UCLA Medical Center, Torrance, CA, USA.

[88]Digital Health Center, Hasso Plattner Institute, University of Potsdam, Prof.-Dr.-Helmert-Str. 2-3, Potsdam, Germany.

[89]Hasso Plattner Institute for Digital Health at Mount Sinai, Icahn School of Medicine at Mount Sinai, One Gustave L. Levy Place, New York, NY, USA.

[90]Department of Human Genetics, McGill University, Montreal, QC, Canada.

[91]Division of Endocrinology and Metabolism, Department of Medicine, McGill University, Montreal, QC, Canada.

[92]McGill University and Génome Québec Innovation Centre, Montreal, QC, Canada.

[93]Department of Twin Research and Genetic Epidemiology, King's College London, London, UK.

[94]Institute for Medical Informatics Biometry and Epidemiology, Ludwig-Maximilians University, Munich, Germany.

[95]Institute of Human Genetics, Helmholtz Zentrum München, German Research Center for Environmental Health, Neuherberg, Germany.

[96]Life Sciences Institute, National University of Singapore, Singapore, Singapore.

[97]Department of Biochemistry, Faculty of Medicine, Health Science Center, Kuwait University, Safat, Kuwait.

[98]Faculty of Medicine, Macau University of Science & Technology, Macau, China.

[99]Department of Pathology and Laboratory Medicine, The Robert Larner M.D. College of Medicine, University of Vermont, Burlington, VT, USA.

[100]Department of Biochemistry, The Robert Larner M.D. College of Medicine, University of Vermont, Burlington, VT, USA.

[101]Department of Endocrinology, Abdominal Centre, Helsinki University Hospital, Helsinki, Finland; Institute for Molecular Medicine Finland, University of Helsinki, Helsinki, Finland

[102]Folkhaalsan Research Centre, Helsinki, Finland; Research Programs Unit, Clinical and Molecular Medicine, University of Helsinki, Helsinki, Finland.

[103]Diabetes Prevention Unit, National Institute for Health and Welfare, Helsinki, Finland.

[104]Center for Vascular Prevention, Danube University Krems, Krems, Austria.

[105]Diabetes Research Group, King Abdulaziz University, Jeddah, Saudi Arabia.

[106]Instituto de Investigacion Sanitaria del Hospital Universario LaPaz (IdiPAZ), University Hospital LaPaz, Autonomous University of Madrid, Madrid, Spain.

[107]Unidad de Biología Molecular y Medicina Genómica, Instituto Nacional de Ciencias Médicas y Nutrición Salvador Zubirán, Mexico City, Mexico.

[108]Departamento de Medicina Genómica y Toxiología Ambiental, Instituto de Investigaciones Biomédicas, UNAM, Mexico City, Mexico.

[109]Department of Nutrition, Harvard T.H. Chan School of Public Health, Boston, MA, USA.

[110]Department of Physiology and Biophysics, University of Mississippi Medical Center, Jackson, MS, USA.

[111]Department of Public Health, Aarhus University, Aarhus, Denmark.

[112]Danish Diabetes Academy, Odense, Denmark.

[113]Boston Children‚Äôs Hospital, Boston, Massachusetts, USA.

[114]Diabetes Unit and Center for Genomic Medicine, Massachusetts General Hospital, Boston, MA, USA.

[115]Genentech, South San Francisco, California, USA.

Supplementary References

1. Emdin CA*, et al.* Genetic Association of Waist-to-Hip Ratio With Cardiometabolic Traits, Type 2 Diabetes, and Coronary Heart Disease. *JAMA* **317**, 626-634 (2017).

2. Ferkingstad E*, et al.* Large-scale integration of the plasma proteome with genetics and disease. *Nat Genet* **53**, 1712-1721 (2021).
