# Supplementary material for: Rare loss of function variants in the hepatokine gene INHBE protect from abdominal obesity
Source: Nat Commun. 2022 Jul 27;13:4319. doi: 10.1038/s41467-022-31757-8 (PMC9329324; doi:10.1038/s41467-022-31757-8)
Supplement: Supplementary file 3 — Description of Additional Supplementary Files [file 41467_2022_31757_MOESM3_ESM.pdf]

Description of Additional Supplementary Files

File Name: Supplementary Data 1
Description: **Expression of WHRadjBMI-associated genes in GTEx.** P-values are from a one-sided Fisher's exact test.

File Name: Supplementary Data 2
Description: **Multi-ancestry analysis of WHRadjBMI in UKB.** Burden testing was performed using a generalized linear model in the other sub-populations and subsequent meta-analysis was performed for variant sets with ≥ 5 carriers in at least one other sub-population. Note: *ACVR1C* pLOF+missense variants were not tested because no pLOF carriers were present in the other sub-populations

File Name: Supplementary Data 3
Description: **Meta-analysis of WHRadjBMI in UKB and AMP-T2D-GENES dataset.** Meta-analysis was performed using an inverse-variance weighted method implemented in METAL and heterogeneity was tested using Cochran's Q test.

File Name: Supplementary Data 4
Description: **Leave-one-variant-out analysis for all significant genes.** Gene-based burden analysis of WHRadjBMI was performed using a generalized linear model excluding one variant at a time from the analysis.

File Name: Supplementary Data 5
Description: **Association of *INHBE* pLOF with quantitative traits.** Association testing was performed using a generalized linear model adjusting for age, sex and 30 PCs of genetic ancestry.

File Name: Supplementary Data 6
Description: **Association of *INHBE* pLOF with circulating proteins.** Association testing was performed using a generalized linear model adjusting for age, sex and 30 PCs of genetic ancestry.

File Name: Supplementary Data 7
Description: **Association of *INHBE* pLOF with disease diagnoses.** Association testing was performed using a mixed-effects model adjusting for age, sex, 30 PCs of genetic ancestry, the availability of primary care data, and country of recruitment.

File Name: Supplementary Data 8
Description: **Association of *INHBE* pLOF with biomarkers from primary care data.** Association testing was performed using a generalized linear model adjusting for age, sex and 30 PCs of genetic ancestry.

File Name: Supplementary Data 9
Description: **Association of *INHBE* pLOF with body composition assessed by DEXA imaging and bioelectrical impedance.** Association testing was performed using a generalized linear model adjusting for age, sex and 30 PCs of genetic ancestry.

File Name: Supplementary Data 10
Description: **Age distribution of *INHBE* pLOF carriers in gnomAD v2.1.1.** The genomic coordinates (GRCh37) as each pLOF variant is supplied along with the number of carriers in each age bin.

File Name: Supplementary Data 11
Description: **Association of Mendelian lipodystrophy genes with WHRadjBMI.** Association testing was performed using a generalized linear model adjusting for 30 PCs of genetic ancestry.

File Name: Supplementary Data 12
Description: **PheWAS of other WHRadjBMI genes.** We performed PheWAS on the most significant variant set per gene. Association testing was performed using a generalized linear model for quantitative traits and a mixed-effects model for disease diagnoses. All significant associations ($P \leq 5\times10^{-5}$) are shown.

File Name: Supplementary Data 13
Description: **Details of AMP-T2D-GENES cohorts.** Baseline characteristics of the cohorts and accession numbers (dbGAP/EGA) for accessing the data are provided.
