# Supplementary material for: Rare loss of function variants in the hepatokine gene INHBE protect from abdominal obesity
Source: Nat Commun. 2022 Jul 27;13:4319. doi: 10.1038/s41467-022-31757-8 (PMC9329324; doi:10.1038/s41467-022-31757-8)
Supplement: Supplementary file 5 — Reporting Summary [file 41467_2022_31757_MOESM5_ESM.pdf]

nature portfolio

Corresponding author(s): Aimee Deaton

Last updated by author(s): Jun 8, 2022

Reporting Summary

Nature Portfolio wishes to improve the reproducibility of the work that we publish. This form provides structure for consistency and transparency in reporting. For further information on Nature Portfolio policies, see our Editorial Policies and the Editorial Policy Checklist.

Statistics

For all statistical analyses, confirm that the following items are present in the figure legend, table legend, main text, or Methods section.

n/a	Confirmed	
☐	☒	The exact sample size (*n*) for each experimental group/condition, given as a discrete number and unit of measurement
☐	☒	A statement on whether measurements were taken from distinct samples or whether the same sample was measured repeatedly
☐	☒	The statistical test(s) used AND whether they are one- or two-sided<br>*Only common tests should be described solely by name; describe more complex techniques in the Methods section.*
☐	☒	A description of all covariates tested
☐	☒	A description of any assumptions or corrections, such as tests of normality and adjustment for multiple comparisons
☐	☒	A full description of the statistical parameters including central tendency (e.g. means) or other basic estimates (e.g. regression coefficient) AND variation (e.g. standard deviation) or associated estimates of uncertainty (e.g. confidence intervals)
☐	☒	For null hypothesis testing, the test statistic (e.g. *F*, *t*, *r*) with confidence intervals, effect sizes, degrees of freedom and *P* value noted<br>*Give P values as exact values whenever suitable.*
☒	☐	For Bayesian analysis, information on the choice of priors and Markov chain Monte Carlo settings
☒	☐	For hierarchical and complex designs, identification of the appropriate level for tests and full reporting of outcomes
☐	☒	Estimates of effect sizes (e.g. Cohen's *d*, Pearson's *r*), indicating how they were calculated

Our web collection on statistics for biologists contains articles on many of the points above.

Software and code

Policy information about availability of computer code

Data collection	No software was used for data collection.
Data analysis	All the software used is publicly available. The following software was used: R version 3.6.0 (base functions and additional packages RNOmni, CMplot, survival, eigenstrat), EPACTS software version 3.2.4 (https://github.com/statgen/EPACTS), REGENIE version 2.2.4 (https://github.com/rgcgithub/regenie), METAL (version release date 2011-03-25; http://csg.sph.umich.edu/abecasis/Metal/download/), Ensembl Variant Effect Predictor (VEP) version 95, Whole Genome Sequence Annotator AMI version 0.8.

For manuscripts utilizing custom algorithms or software that are central to the research but not yet described in published literature, software must be made available to editors and reviewers. We strongly encourage code deposition in a community repository (e.g. GitHub). See the Nature Portfolio guidelines for submitting code & software for further information.

Data

Policy information about availability of data

All manuscripts must include a data availability statement. This statement should provide the following information, where applicable:

- Accession codes, unique identifiers, or web links for publicly available datasets
- A description of any restrictions on data availability
- For clinical datasets or third party data, please ensure that the statement adheres to our policy

For the primary analysis in UKB, phenotypic data, exome sequencing and whole genome sequencing data are accessible through application to UKB with the exception of proteomic data which is scheduled for release in October 2022. For replication analysis in AMP-T2D-GENES, details on how to access results from the individual studies can be found in the original publications (refs 29, 68, 69, 70, 71) and a list of dbGAP/EGA accession numbers is provided in Supplementary Data

13. Additional data is publicly available through the T2D Knowledge Portal at https://t2d.hugeamp.org/dinspector.html?dataset=ExSeq_52kQT. gnOMAD data is available for download at https://gnomad.broadinstitute.org/downloads. Source data are provided with this paper.

Field-specific reporting

Please select the one below that is the best fit for your research. If you are not sure, read the appropriate sections before making your selection.

☒ Life sciences ☐ Behavioural & social sciences ☐ Ecological, evolutionary & environmental sciences

For a reference copy of the document with all sections, see nature.com/documents/nr-reporting-summary-flat.pdf

Life sciences study design

All studies must disclose on these points even when the disclosure is negative.

Sample size	Sample size was not predetermined and the maximum sample size available in the UK Biobank dataset was used. Power calculations were later performed where required.
Data exclusions	Subject and variant quality control was performed as described in the methods. All data passing quality control were included in the analysis. Genetic associations were performed within sub-populations defined by genetic ancestry.
Replication	Replication of genetic findings was performed in the AMP-T2D-GENES dataset. For in vitro experiments, three biological replicates were performed and all attempts at replication were successful.
Randomization	Association testing in UK Biobank was performed across all unrelated individuals, stratified by genetic ancestry. For analysis of WHRadjBMI, values were adjusted for age and sex prior to association testing. In the association analysis the covariates used were PCs 1-30 of genetic ancestry. Association testing in AMP-T2D-GENES was performed across all unrelated individuals with WHRadjBMI measurements available in a single "mega-analysis". Covariates used were PCs 1-10 of genetic ancestry, sample cohort subgroup and sequencing technology.<br>For experiments characterizing INHBE variants in vitro, for each replicate, the same passage of HEK293T cells was used for transfection of the various expression constructs so randomization was not relevant. For expression analysis, samples were taken from separate cohorts of lean and obese monkeys whose differences are part of the biology we are studying (age, diet, weight) so randomization was not possible.
Blinding	Blinding was not relevant for the genetic association testing as all individuals were analyzed as described above (and not allocated to separate groups). Blinding was not feasible for the in vitro variant characterization and expression analysis as a single individual conducted each experiment. Blinding is not necessary for these experiments as the results are quantitative and do not require subjective interpretation.

Reporting for specific materials, systems and methods

We require information from authors about some types of materials, experimental systems and methods used in many studies. Here, indicate whether each material, system or method listed is relevant to your study. If you are not sure if a list item applies to your research, read the appropriate section before selecting a response.

Materials & experimental systems

n/a	Involved in the study	
☐	☒	Antibodies
☐	☒	Eukaryotic cell lines
☒	☐	Palaeontology and archaeology
☐	☒	Animals and other organisms
☐	☒	Human research participants
☒	☐	Clinical data
☒	☐	Dual use research of concern

Methods

n/a	Involved in the study	
☒	☐	ChIP-seq
☒	☐	Flow cytometry
☒	☐	MRI-based neuroimaging

Antibodies

Antibodies used	Anti-FLAG antibody (1:6000; Monoclonal Anti-FLAG M2 catalog number F3165, MilliporeSigma, Burlington, MA)<br>Anti-βactin (1:8000; Monoclonal Anti-βactin AC-15, catalog number A5441, MilliporeSigma, Burlington, MA)<br>IRDye® 680RD Goat anti-Mouse IgG Secondary Antibody (1:10,000; catalog number P/N 925-68070 LI-COR, Lincoln, NE)
Validation	Validation statements are available on the manufacturers' website. Relevant information below:<br>Anti-FLAG: species reactivity all; techniques western blot<br>Anti-βactin: species reactivity includes human; techniques include western blot at 1:5,000-1:10,000<br>IRDye® 680RD Goat anti-Mouse IgG Secondary Antibody: Odyssey western blot detection at 1:5,000-1:25,000

Eukaryotic cell lines

Policy information about cell lines

Cell line source(s)	HEK293T cells (CRL-3216 ATCC; Manassas, VA)
Authentication	Cell line was not authenticated.
Mycoplasma contamination	Cell line was not tested for mycoplasma contamination.
Commonly misidentified lines (See ICLAC register)	Cell line is not commonly misidentified.

Animals and other organisms

Policy information about studies involving animals; ARRIVE guidelines recommended for reporting animal research

Laboratory animals	Lean cynomolgus monkeys (Macaca fascicularis, age 2-4 years, weight 1.9-6.0kg, male and female) and cynomolgus monkeys on a high fat diet displaying features of non-alcoholic steatohepatitis (Macaca fascicularis, age > 8 years, weight > 7.0kg, male).
Wild animals	No wild animals were used in this study
Field-collected samples	No field-collected samples were used in this study
Ethics oversight	Ethical approval was obtained from the Charles River Laboratory Institutional Animal Care and Use Committee (IACUC) and the Kunming Biomed International IACUC.

Note that full information on the approval of the study protocol must also be provided in the manuscript.

Human research participants

Policy information about studies involving human research participants

Population characteristics	Participants in UK Biobank have been described previously (https://doi.org/10.1093/aje/kwx246). Participants were ~500,000 healthy volunteers aged between 40 and 69 at enrollment. The cohort was 54% female and 46% male.
Recruitment	Recruitment for UK Biobank and the biases resulting have been described by UK Biobank and in the literature (for example https://doi.org/10.1093/aje/kwx246). Volunteers are more likely to be female and in better health than the overall population.
Ethics oversight	UK Biobank study was approved by the National Health Service National Research Ethics Service and all participants provided written informed consent to participate in the study. The UK Biobank resource is an approved Research Tissue Bank and is registered with the Human Tissue Authority, which means that researchers who wish to use it do not need to seek separate ethics approval. This research has been conducted using the UK Biobank resource, applications 26041 and 65851.<br><br>All individuals in the AMP-T2D-GENES study provided informed consent and all samples were approved for use at the respective institution.

Note that full information on the approval of the study protocol must also be provided in the manuscript.
